# External treatment of traditional Chinese medicine for COVID-19: A protocol for systematic review and meta-analysis

**DOI:** 10.1097/MD.0000000000022316

**Published:** 2020-09-25

**Authors:** Liu Wu, Qiang Yuan, Yongbing Kuang, Yong Chen, Jin Li, Yinhao Feng, Jian Luo

**Affiliations:** a Department of Tuina, Hospital of Chengdu University of Traditional Chinese Medicine, Chengdu, Sichuan Province, P. R. China; b Emergency Department, Hospital of Chengdu University of Traditional Chinese Medicine, Chengdu, Sichuan Province, P. R. China.

**Keywords:** COVID-19, systematic review, traditional Chinese medicine

## Abstract

**Background:**

There is a worldwide outbreak of COVID-19, as the number of patients increases. External treatment of traditional Chinese medicine includes acupuncture, massage, fire needle, cupping, and other alternative therapies. Currently, there are no relevant articles for systematic review.

**Methods:**

We will search the randomized controlled trials related to the external treatment of traditional Chinese medicine (such as, acupuncture, massage, etc) and COVID-19 from inception to June 2020. The following database is our focus area: the Cochrane Central Register of Controlled Trials (CENTRAL), PubMed, EMBASE, Web of Science, China National Knowledge Infrastructure, Chinese Biomedical Literature Database, and Wan-Fang Database. All published randomized controlled trials in English or Chinese related to massage for COVID-19 will be included. Primary outcomes include the influence of external treatment of traditional Chinese medicine on the patients with COVID-19. Secondary outcomes include accompanying symptoms (such as myalgia, expectoration, stuffiness, runny nose, pharyngalgia, anhelation, chest distress, dyspnea, crackles, headache, nausea, vomiting, anorexia, diarrhea) disappear rate, negative COVID-19 results rate on 2 consecutive occasions (not on the same day), average hospitalization time, Clinical curative effect, and improved quality of life.

**Results:**

The results will provide a high-quality synthesis of current evidence for researchers in this subject area.

**Conclusion:**

The conclusion of our study will provide evidence to judge whether external treatment of traditional Chinese medicine is an effective intervention on the patients with COVID-19.

**PROSPERO registration number:**

CRD42020181336

## 1 Introduction

At the end of 2019, a series of pneumonia cases of unknown cause emerged in Wuhan (Hubei, China).^[1]^ A few weeks later, in January 2020, deep sequencing analysis from lower respiratory tract samples identified a novel virus severe acute respiratory syndrome coronavirus 2 (SARS-CoV-2) as a causative agent for that observed pneumonia cluster.^[2]^ On February 11th, 2020, the World Health Organization (WHO) Director-General, Dr. Tedros Adhanom Ghebreyesus, named the disease caused by the SARS-CoV-2 as “COVID-19,” and by May 6th, 2020 when the number of countries involved was 211 with more than 3,700,000 cases and over 260,000 deaths, the WHO declared the pandemic status.

Through the analysis of sequence, this unidentified pneumonia was considered to be caused by a novel coronavirus (CoV) named 2019-nCoV.^[3]^ Subsequently, the WHO announced a standard format of Coronavirus Disease-2019 (COVID-19), according to its nomenclature, for this novel coronavirus pneumonia on February 11, 2020.^[4]^ On the same day, the International Committee on Taxonomy of Viruses (ICTV) named this novel coronavirus as SARS-CoV-2.^[5]^ So far, the SARS-CoV-2 infection is still spreading, and this virus poses a serious threat to public health, though joint prevention and quarantine mechanisms in almost all provinces of mainland China have been confirmed to be enacted. Due to a lack of specific antiviral treatments and pressure of clinical treatment, thousands of severe cases have died every day worldwide.

The patients also displayed various clinical symptoms, such as fever (83%), cough (82%), shortness of breath (31%), muscle ache (11%), confusion (9%), headache (8%), sore throat (5%), rhinorrhea (4%), chest pain (2%), diarrhea (2%), and nausea and vomiting (1%) (see Fig. 5B). Approximately 90% of the patients were observed to have more than one of the symptoms, whereas 15% experienced fever, cough, and shortness of breath simultaneously.^[6]^

The outbreak of emerging severe acute respiratory syndrome coronavirus 2 (SARS-CoV-2) disease (COVID-19) in China has been brought to global attention and declared a pandemic by the WHO on March 11, 2020. Initial epidemiological investigation suggested that a majority of suspected cases were associated with their presence (exposure) in a local Huanan seafood market. However, such a decisive conclusion was disputed because the earliest case had had no reported link connection to the mentioned market.^[7]^ In addition, it was found that at least 2 different strains of SARS-CoV-2 had occurred a few months earlier before COVID-19 was officially reported.^[8]^ A recently phyloepidemiologic analysis suggests that SARS-CoV-2 at the Huanan Seafood Market could have been imported from other places.^[9]^

Currently, COVID-19 patients are the main source of infection, and severe patients are considered to be more contagious than mild ones. Asymptomatically infected persons or patients in incubation who show no signs or symptoms of respiratory infection proven to shed infectious virus, may also be potential sources of infection.^[10]^ In addition, samples taken from patients recovered from COVID-19 continuously show a positive RT-PCR test,^[11]^ which has never been seen in the history of human infectious diseases. In other words, asymptomatically infected persons and patients in incubation or recovered from COVID-19 may pose serious challenges for disease prevention and control.

External treatment of traditional Chinese medicine does not refer to a special treatment, but refers to acupuncture, moxibustion, massage and a series of external treatment. The acupuncture and moxibustion have been practiced in China for more than 3000 years and was spread to Europe and American from the sixteenth to the nineteenth century.^[12]^ The history of acupuncture research was initiated in the eighteenth century and developed rapidly since then. Accumulated evidences that acupuncture and moxibustion are beneficial in various conditions significantly enhanced our understanding the mechanisms of acupuncture treatment.

Acupuncture is a component of External treatment of traditional Chinese medicine. According to the literature, acupuncture can enhance the immune system function of patients and thus play a role In the treatment of respiratory diseases.^[13]^ According to a number of clinical practices, acupuncture therapy has a considerable effect, has a high technical content, can inhibit allergic reactions, activate its defense system function, and the patient's bronchial smooth muscle tone is reduced, and finally eliminate a part of respiratory symptoms.^[14]^ COVID-19 is a respiratory disease, so acupuncture may be effective in the treatment of COVID-19, especially in the improvement of some related symptoms.

Massage is also a kind of external treatment of traditional Chinese medicine; it is a preventive and restorative therapy involving the systematic application of pressure to the skin, muscle, and connective tissue with the aim of improving blood and lymph circulation.^[15]^ With the outbreak of the epidemic, COVID-19 is not only harmful for our body but also for the mental health, especially the people who have recovered from covid-19. Massage has been shown to relieve psychological problems such as anxiety, insomnia, depression aggression, frustration, and hysteria.^[16]^

Currently, there is still a lack of evidence-based medical evidence for the external treatment of traditional Chinese medicine for COVID-19. Therefore, it is necessary to review it and provide evidence for clinicians.

## 2 Methods

### 2.1 Study registration

The systematic review protocol has been registered in PROSPERO. The registration number: CRD42020181336, the consent of this protocol report is based on the Preferred Reporting Items for Systematic Reviews and Meta-Analyses Protocols (PRISMAP) statement guidelines.^[17]^

### 2.2 Inclusion criteria for study selection

#### 2.2.1 Type of study

We will include articles related to the external treatment of traditional Chinese medicine (such as, acupuncture, massage, etc) of patients with COVID-19. Due to language restrictions, we will search for articles in English and Chinese in order to get a more objective and true evaluation; all articles included are randomized controlled trial (RCT) type articles.

#### 2.2.2 Type of participant

All patients with COVID-19 will be included regardless of sex, age, race, education, and economic status. Pregnant women, postoperative infections, psychopaths, and patients with severe cardiovascular and liver and kidney diseases will not be included.

#### 2.2.3 Type of intervention

The external treatment of traditional Chinese medicine (such as, acupuncture, massage, etc) while other traditional Chinese therapies will be excluded. We will compare the following interventions: treatments other than the external treatment of traditional Chinese medicine (eg, usual or standard care, placebo, wait-list controls).

#### 2.2.4 Type of outcome measure

Primary outcomes include the influence of the external treatment of traditional Chinese medicine on patients with COVID-19. Secondary outcomes include accompanying symptoms (such as myalgia, expectoration, stuffiness, runny nose, pharyngalgia, anhelation, chest distress, dyspnea, crackles, headache, nausea, vomiting, anorexia, diarrhea) disappear rate, average hospitalization time, occurrence rate of common type to severe form, clinical cure rate, and mortality.

### 2.3 Data sources

The following electronic databases will be searched from inception to May 2020: the Cochrane Central Register of Controlled Trials (CENTRAL), PubMed, EMBASE, Web of Science, China National Knowledge Infrastructure, Chinese Biomedical Literature Database, and Wan-Fang Database. About other sources, we also plan to manually search for the unpublished conference articles and the bibliography of established publications.

### 2.4 Search strategy

The search terms on PubMed are as follows: massage (eg, “acupoints” or “tuina” or “manipulation”); acupuncture (eg, “acupuncture” or “acupuncture therapy” or “body acupuncture” or “manual acupuncture” or “electroacupuncture” or “fire needling” or “plum blossom needling”); COVID-19 (eg, “Corona Virus Disease 2019” or “Corona Virus”); RCT (eg, “randomized” or “randomly” or “clinical trial”). Combinations of Medical Subject Headings (MeSH) and text words will be used. The same search term is used in electronic databases in China. These search terms are summarized in Table 1.

**Table 1:**
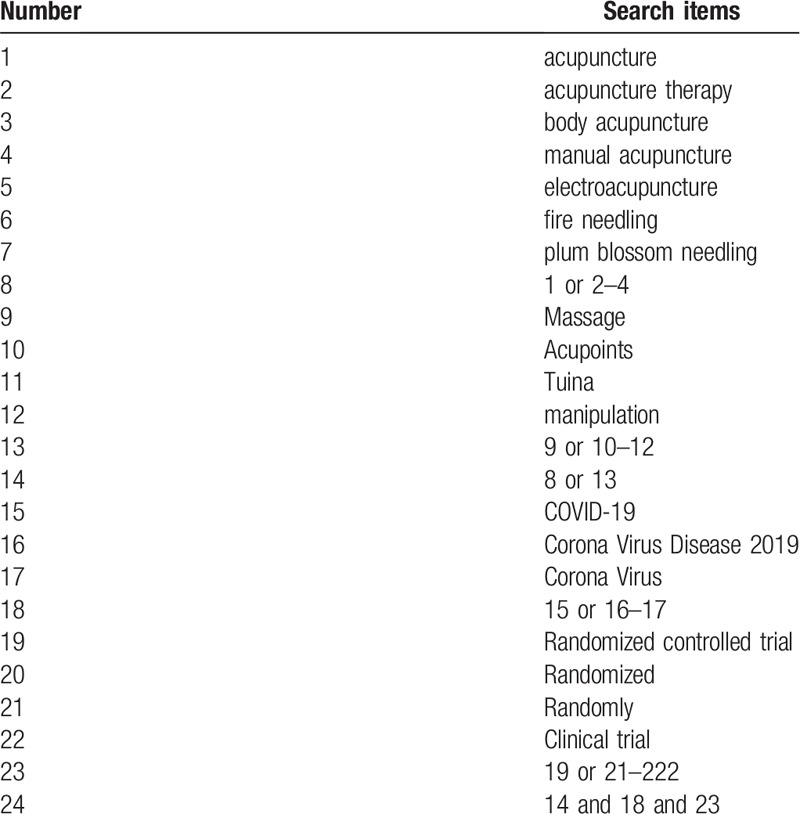
Search strategy for the PubMed database.

### 2.5 Data collection and analysis

#### 2.5.1 Selection of studies

**Figure 1:**
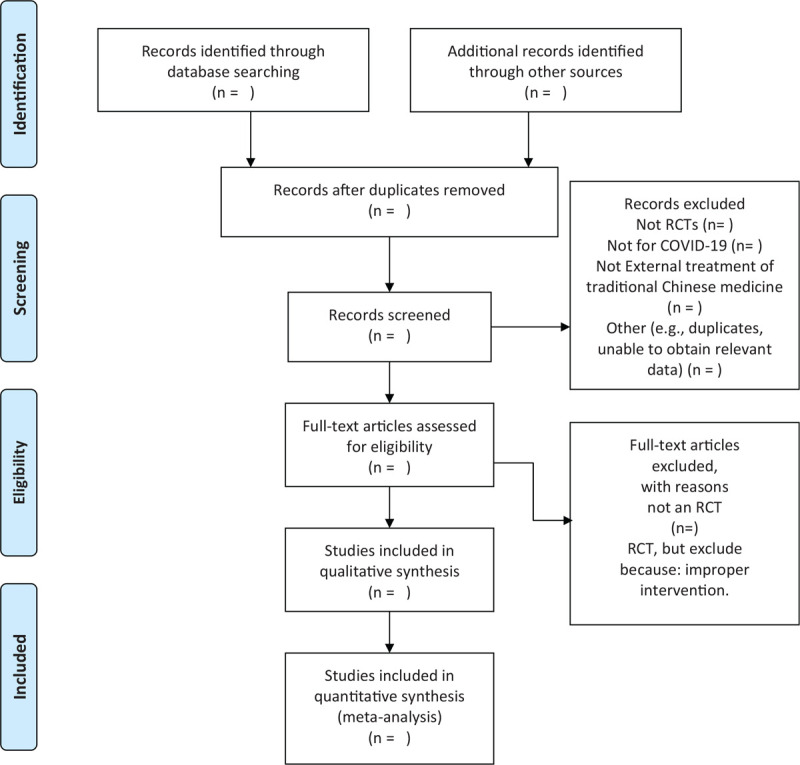
Flow chart of the study.

We chose the PRISMA flow chart to show the process of selecting literature for the entire study (Fig. 1). Before searching the literature, all reviewers will discuss and determine the screening criteria. After the screening requirements are clearly defined, the 2 reviewers will independently review and screen the literature. They screened the titles and abstracts of the literature, in order to get qualified studies, and then excluded some duplicate studies or studies with incomplete information. We will also try to obtain the full text, and the obtained literature will be managed by using EndNote software, V.X8 (United States). In case of disagreement between the 2 reviewers, discussions will be held with the third author for arbitration.

#### 2.5.2 Data extraction and management

The authors will strictly follow the inclusion criteria and select RCT articles related to the topic. Through the analysis of the article, we know participants’ characteristics (height, weight, sex), interventions, outcomes, the study characteristics (press, nationality, journals, research design), adverse reactions, etc. If there is any disagreement between the 2 authors in the literature data extraction, a third article participant will discuss the decision together. If there is a lack of data in the literature, we will contact the author or publisher as much as possible.

#### 2.5.3 Assessment of risk of bias in included studies

We will use the Cochrane collaborative tool to independently assess the risk of bias in the included studies. We will evaluate the following aspects of the article: sequence generation, assignment sequence hiding, blindness of participants and staff, outcome evaluators, incomplete result data, selective result reporting, and other sources of bias. The risk of bias is evaluated at 3 levels, namely, low risk, high risk, and ambiguity. If the information is vague, we will try to contact the author of the article.

#### 2.5.4 Measures of treatment effect

In this protocol, we will use 95% confidence interval (95% CI) risk ratio (RR) to rigorously analyze the dichotomous data. And for the continuous data, mean difference (MD) or standard MD (SMD) is used to measure the efficacy of 95% CI.

#### 2.5.5 Unit of analysis issues

We will include data from parallel group design studies for meta-analysis. In these trials, we will collect and analyze individual measurements of each outcome for each participant.

#### 2.5.6 Management of missing data

We will try our best to ensure the integrity of the data. If the included RCT data is not complete, we will try every means to contact the corresponding author of the article, including sending emails or making a phone call. If the corresponding author cannot be contacted, we will remove the experiment with incomplete data. After data integrity is assured, intention analysis therapy and sensitivity analysis will be performed.

#### 2.5.7 Assessment of heterogeneity

For the detection of heterogeneity, the *I*^2^ test will be used to detect the heterogeneity among trials. When the *I*^2^ test value is <50% and *P* > 1, we think there is no heterogeneity between these trials, and when the *I*^2^ test value is >50% and the *P* value is <1, there is significant heterogeneity between these included trials. If significant differences are detected, we will analyze the possible causes of heterogeneity, and then we can use the random effects model.

#### 2.5.8 Assessment of reporting biases

In this analysis, once >10 trials are included, funnel plots could be used to test for reporting bias.

#### 2.5.9 Data synthesis

We will use Review Manager Software (RevMan) V.5.3 (Copenhagen, Denmark) for data analysis and quantitative data synthesis. If there is no finding of statistical heterogeneity, the fixed-effect model is used for data synthesis. If there is significant statistical heterogeneity, we will use the random effect model, and all participants will explore the possible causes from a clinical and methodological perspective and provide a descriptive or subgroup analysis.

#### 2.5.10 Subgroup analysis

Subgroup analysis will be performed to explain heterogeneity if possible. Factors such as different types of control interventions and different outcomes will be considered.

#### 2.5.11 Sensitivity analysis

On the basis of sample size, study design, heterogeneous quality, methodological quality, and statistical model, sensitivity analysis will be performed to exclude trials with quality defects and ensure the stability of the analysis results.

#### 2.5.12 Grading the quality of evidence

This paper will use the evidence quality rating method to evaluate the results obtained from this analysis. GRADE is generally applied to a large amount of evidence. It has 4 evaluation levels, namely, high, medium, low, and very low. GRADE was used to evaluate the bias, inconsistencies, discontinuities, and inaccuracies of test results. In the context of the system review, quality reflects our confidence in the effectiveness of assessment.^[17]^

#### 2.5.13 Ethical review and informed consent of patients

The content of this article does not involve moral approval or ethical review and will be presented in print or at relevant conferences.

## 3 Discussion

This review is divided into 4 parts: identification, literature inclusion, data extraction, and data synthesis. It will systematically review the RCT literature; this review will evaluate the effectiveness of the external treatment of traditional Chinese medicine in treating COVID-19. There are also limitations in our research and the language bias here is that we only search for Chinese and English documents. Our study may provide a basis for clinicians to choose replacement therapy for further study in the future.

## Author contributions

**Conceptualization:** Liu Wu, Qiang Yuan.

**Data curation:** Qiang Yuan, Liu Wu, Yongbing Kuang.

**Formal analysis:** Yinhao Feng, Qiang Yuan, Jin Li, Yong Chen.

**Funding acquisition:** Jian Luo.

**Investigation:** Liu Wu, Yongbing Kuang.

**Methodology:** Liu Wu, Yongbing Kuang.

**Project administration:** Yong Chen.

**Resources:** Liu Wu, Jian Luo.

**Software:** Liu Wu.

**Supervision:** Yongbing Kuang, Jin Li.

**Validation:** Jin Li.

**Visualization:** Yinhao Feng.

**Writing – original draft:** Qiang Yuan, Liu Wu.

**Writing – review & editing:** Qiang Yuan, Liu Wu, Yongbing Kuang.
